# Moral Distress and Perceived Community Views Are Associated with Mental Health Symptoms in Frontline Health Workers during the COVID-19 Pandemic

**DOI:** 10.3390/ijerph18168723

**Published:** 2021-08-18

**Authors:** Natasha Smallwood, Amy Pascoe, Leila Karimi, Karen Willis

**Affiliations:** 1 Department of Respiratory Medicine, The Alfred Hospital, Prahan, VIC 3004, Australia; 2 Department of Allergy, Immunology and Respiratory Medicine, Central Clinical School, The Alfred Hospital, Monash University, Melbourne, VIC 3004, Australia; amy.pascoe@svha.org.au; 3 School of Psychology and Public Health, La Trobe University, Melbourne, VIC 3083, Australia; l.karimi@latrobe.edu.au; 4 School of Medicine and Healthcare Management, Caucasus University, Tbilisi 0102, Georgia; 5 School of Public Health, College of Health and Biomedicine, Victoria University, Footscray Park, Melbourne, VIC 3011, Australia; karen.willis@vu.edu.au; 6 Division of Critical Care and Investigative Services, Royal Melbourne Hospital, Grattan Street Parkville, Melbourne, VIC 3050, Australia

**Keywords:** COVD-19, moral distress, healthcare worker, mental health, communication, leadership

## Abstract

Background: Sudden changes in clinical practice and the altered ability to care for patients due to the COVID-19 pandemic have been associated with moral distress and mental health concerns in healthcare workers internationally. This study aimed to investigate the severity, prevalence, and predictors of moral distress experienced by Australian healthcare workers during the COVID-19 pandemic. Methods: A nationwide, voluntary, anonymous, single time-point, online survey of self-identified frontline healthcare workers was conducted between 27th August and 23rd October 2020. Participants were recruited through health organisations, professional associations, or colleges, universities, government contacts, and national media. Results: 7846 complete responses were received from nurses (39.4%), doctors (31.1%), allied health staff (16.7%), or other roles (6.7%). Many participants reported moral distress related to resource scarcity (58.3%), wearing PPE (31.7%) limiting their ability to care for patients, exclusion of family going against their values (60.2%), and fear of letting co-workers down if they were infected (55.0%). Many personal and workplace predictors of moral distress were identified, with those working in certain frontline areas, metropolitan locations, and with prior mental health diagnoses at particular risk of distress. Moral distress was associated with increased risk of anxiety, depression, post-traumatic stress disorder, and burnout. Conversely, feeling appreciated by the community protected against these risks in healthcare workers. Conclusions: Safeguarding healthcare workforces during crises is important for both patient safety and workforce longevity. Targeted interventions are required to prevent or minimise moral distress and associated mental health concerns in healthcare workers during COVID-19 and other crises.

## 1. Introduction

The COVID-19 pandemic has led to growing international awareness regarding the prevalence of moral distress and psychological symptoms amongst frontline healthcare workers (HCWs) [1,2]. Moral distress, also referred to as ‘moral injury’, is defined as ‘perpetrating, failing to prevent, bearing witness to, or learning about acts that transgress deeply help moral beliefs and expectations’ [3] (page 695). Moral distress is known to arise from situations that prevent HCWs from delivering care in the way they have been trained [4,5,6]. Systemic problems within the healthcare system that impact patient care such as scarcity of resources, inadequately preventing harm or death, and failing to meet patients’ needs can be morally challenging for HCWs [4,5,6]. Such situations have arisen both during previous public health events, such as SARS [7], H1N1 influenza [8], and Ebola epidemics [9], as well as the current COVID-19 pandemic [10]. In addition to moral distress, HCWs exhibit high rates of mental health problems, both in non-pandemic times and during the current COVID-19 pandemic [11,12,13]. Experiencing moral distress can be a contributing or compounding factor in the development of broader mental health problems [4,13]; burnout in particular has been linked to moral distress [14] and has adverse impacts on patient care [15] and job satisfaction [16]. Protecting workers from moral distress and associated burnout, particularly during crises, is an important consideration in workforce retention. Public initiatives to demonstrate community appreciation and gratitude for healthcare workers have been popular throughout COVID-19, with some evidence that positive community perceptions can bolster mental health [17].

While the effects of moral distress seem to be similar to those resulting from psychological distress and burnout, studies examining the relationship between these concepts in frontline HCWs are limited [12,18,19]. This article reports a subset of findings from the Australian COVID-19 Frontline Healthcare Workers’ Study regarding the prevalence and predictors of specific factors of moral distress relating to resource scarcity, patient care, burdening of co-workers, and perceptions of both stigma and appreciation in the broader community during the COVID-19 pandemic, as well as their relationships with mental health outcomes amongst frontline HCWs. Understanding factors of moral distress prevalence and impacts on mental health is important to inform targeted interventions to safeguard frontline workforces during current and future crises.

## 2. Materials and Methods

Frontline HCWs were invited to participate in a nationwide, voluntary, confidential, online survey between 27 August and 23 October 2020. The recruitment period coincided with the second wave of the pandemic in Australia, with most cases arising in Melbourne, in the Australian state of Victoria [20]. Multiple recruitment strategies were utilised. Information regarding the survey was emailed to CEOs and departmental directors of frontline areas (emergency medicine, critical care, respiratory medicine, general medicine, infectious diseases, palliative care, and hospital aged care) of all public hospitals throughout Victoria and to multiple hospitals around Australia. Thirty-six professional societies, colleges, universities, associations, and government health department staff also disseminated information about the survey across Australia. Additionally, the study was promoted through 117 newspapers, 8 television and radio news items, and 30 social media sites.

### 2.1. Data Collection

Participants either directly completed the online survey through organizational invitation or via a public-facing purpose-built website (https://covid-19-frontline.com.au/), which held the online survey link. Online consent was acquired from participants prior to commencing the survey, and each respondent could only participate once. The survey included seven sections (Supplementary Material): demographics, professional background and work arrangements, impact of pandemic on employment and finances, exposure to COVID-19, ‘relaxing and staying healthy’, organisational leadership, and workplace change, and five validated psychological assessment scales tools (the Generalised Anxiety Disorder (GAD-7), Patient Health Questionnaire (PHQ-9), abbreviated Impact of Event Scale (IES-6), abbreviated Maslach Burnout Inventory (MBI), and abbreviated 2-item CD-RISC-2 scale to measure resilience). Most sections contained questions in single and multiple-choice format, with questions comprising a five-point Likert scale and some free text responses. Data were collected and managed using REDCap electronic data capture tools [21]. Two questions examined how participants believed the community viewed frontline workers, and four questions investigated moral distress. To ensure participants were able to directly relate their responses to the COVID-19 pandemic, these questions were generated by drawing on contemporary literature about moral distress [22], key insights about the applicability of moral distress during the COVID-19 pandemic [23], and consensus discussions amongst the research team. Ethics approval was provided by the Royal Melbourne Hospital Human Research Ethics Committee (HREC/67074/MH-2020).

### 2.2. Statistical Methods and Data Analysis

A power calculation for general linear models was computed using RStudio [24]. With an expected medium to large effect size, a power of 0.95, and significance level of 0.05, 6348 participants were required. Data analysis was performed using SPSS statistical software version 26.0 (IBM Corp, Armonk, NY, USA). Data are reported descriptively with frequency counts and percentages. For the regression model, mental health scale outcomes were categorised as follows: MBI depersonalisation: 0–3 low, 4–18 moderate to high; emotional exhaustion: 0–6 low, 7–18 moderate to high; personal accomplishment: 0–13 low; 13–18 moderate to high [25]; IES: 0–9 min/none, >9 mod-severe [26]; GAD-7: 0–9 none/minimal to mild, 10–21 moderate to severe [27]; PHQ-9: 0–9 none/minimal to mild, 10–27 moderate to severe [28]. Predictors of moral distress and associations between moral distress and mental health symptoms were identified through univariate logistic regression, then entered into multivariate logistic regression models. Multivariate models were developed using stepwise selection and backward elimination procedures before undergoing a final assessment for clinical and biological plausibility. Variables with a p value of less than 0.10 on univariable analyses or those deemed to be clinically significant were considered for inclusion in the multivariable models.

Covariates examined in regression analyses for moral distress and perceived community views of HCWs included: age, gender, state, occupation, number of years working since graduating, current employment status, frontline area, practice location, works with COVID-19 patients, close friends/relatives with COVID-19, pre-existing mental health condition, received PPE training, confidence in using PPE, received training to care for COVID-19 patients, confidence in caring for COVID-19 patients, and requires further training with PPE or managing COVID-19 patients. Associations are presented as odds ratios (ORs) with 95% confidence intervals (CIs) with statistical significance declared at *p* < 0.05.

## 3. Results

Most participants were female (6344, 80.9%) with an even spread of age ranges (Table 1). Participants primarily resided in Victoria (6685, 85.2%), with the remainder spread across other Australian states and territories. Most participants were nurses (3222, 39.4%), doctors (2436, 31.1%), or allied health professionals (1314, 16.7%), with the remainder holding administrative (485, 6.2%) or other health roles (523, 6.7%). Almost one third of participants (2389, 30.4%) reported having a pre-existing mental illness diagnosed prior to the pandemic. 

### 3.1. Moral Distress and Perceived Community Views of Healthcare Workers

Most participants somewhat or strongly agreed with statements regarding concerns about patients not receiving care due to scarcity of resources (4568, 58.3%) and excluding family from the bedside of patients infected with COVID-19 went against their values as HCWs (4720, 60.2%; Table 2). A third (2729, 31.7%) felt that wearing PPE limited their ability to care for people with COVID-19, and more than half (4318, 55.0%) indicated they would be letting down their co-workers if required to quarantine. Three quarters of participants (6017, 76.7%) believed that the community was worried that HCWs would spread the virus to others. However, most participants (6784, 86.5%) believed that the community appreciated HCWs during the pandemic.

### 3.2. Predictors of Moral Distress

In the multiple regression model, independent predictors for HCWs being worried that scarcity of resources would limit the care given to COVID-19 patients included working in primary, community, or aged care, ICU (relative to emergency department), working in metropolitan areas (relative to regional or remote), and those with a pre-existing mental health diagnosis (Table 3). Participants who worked as nurses or allied health professionals (compared to doctors) were less likely to worry about resource scarcity.

Independent predictors for being concerned that wearing PPE would limit the care provided to COVID-19 patients included living in the state of Victoria (compared to other states) or currently working with COVID-19 patients (Table 3). Individuals who worked in nursing, allied health, and other health roles (compared to doctors), or who were confident using PPE (compared to not confident) were significantly less worried about the effects of wearing PPE on patient care.

Participants who worked in primary, community care, and aged care (compared to ED), those living in metropolitan areas (compared to regional or remote areas), who had pre-existing mental health diagnoses, or who desired more training regarding PPE or managing patients with COVID-19 (compared to those who did not) were significantly more likely to be worried about burdening their co-workers if they needed to quarantine (Table 3). Nurses and allied health professions (compared to doctors) and people working in ICU (compared to ED) were significantly less likely to worry about the effects of quarantine on co-workers’ caseloads.

Independent predictors for believing that excluding family from the bedside of COVID-19 patients went against their values as HCWs included female gender (relative to male), having a pre-existing mental health diagnosis, and indicating need for more training in care for patients with COVID-19 or use of PPE (Table 3). Non-medical staff (in comparison to doctors) and participants who worked in ICU, anaesthetics and surgery, medical specialties, and other frontline areas (in comparison to ED) were significantly less likely to worry about excluding family from the bedside of COVID-19 patients.

### 3.3. Predictors of Perceived Community Views Regarding HCWs

Older participants (compared to those aged 20–30 years) were significantly more likely to believe that the community was worried that HCWs would spread the virus to others (Table 3). People who worked in ‘other’ non-medical roles (including paramedicine, radiology, pharmacy, pathology, maintenance, clerical and admin staff, and COVID-19 screening) compared to doctors were 47% less likely to believe that the community was worried HCWs would spread the virus.

Independent predictors for believing that the community was appreciative of HCWs during the pandemic included: age, occupation, receiving PPE training, and being confident using PPE. Participants aged 31–40 years (compared to aged 20–30 years) and nurses (compared with doctors) were significantly less likely to believe the community was appreciative of HCWs during the pandemic. Participants who were confident using PPE, trained in using PPE, and participants from other health roles (compared to doctors) were more likely to believe the community was appreciative of HCWs during the pandemic.

### 3.4. Relationship between Moral Distress, Perceived Community Views, and Mental Health Outcomes

Being concerned that wearing PPE affected their ability to care for patients with COVID-19 or being worried about letting down colleagues if they needed to quarantine were significant, independent predictors for adverse mental health outcomes on all scales except the personal achievement domain of the burnout scale (Table 4). Being worried about excluding family members from COVID-19 patients’ bedsides was also a significant, independent predictor for all adverse mental health outcomes except depersonalisation and personal achievement. Worrying that patients would not receive appropriate care due to scarcity of healthcare resources was a significant independent predictor for experiencing PTSD and both the emotional exhaustion and depersonalisation domains of burnout. Believing that the community was concerned that HCWs would spread COVID-19 to other people was a significant, independent predictor for experiencing anxiety, PTSD, and both the emotional exhaustion and depersonalisation domains of burnout. Believing that the community was appreciative of HCWs during the pandemic was a significant, independent predictor for experiencing fewer mental health symptoms on all scales and greater personal accomplishment. 

## 4. Discussion

To our knowledge, this is the largest, multi-professional study globally to investigate moral distress and mental health outcomes in frontline HCWs during the COVID-19 pandemic. We have identified personal and work-related predictors for experiencing certain facets of moral distress, as well as demonstrating that prior mental illness, working in primary care, community or aged care, working in metropolitan areas, and desiring more training were predictors of experiencing moral distress. Experiencing moral distress was associated with broad, adverse mental health outcomes, whereas believing that that the community was appreciative of healthcare workers during the pandemic protected against these outcomes. Consistent with previous studies [6,19], our findings suggest that moral distress could be not only a predictor for, but a compounding factor in, the presentation of mental illness symptoms.

### 4.1. Prevalence and Predictors of Moral Distress and Community Perceptions

Participants were broadly in agreement with three of the four indicators of moral distress relating to resource scarcity, exclusion of family, and perceived letting down of overstretched co-workers if quarantined. Fewer participants agreed that PPE usage limited ability to care for patients with COVID-19, though this was still a concern for a third of participants. Although most agreed that the community was concerned about HCWs spreading the virus, the vast majority felt that the community was appreciative of HCWs.

Overall, those with prior mental health diagnoses, primary, community, and aged care health workers, those living in metropolitan areas, and those who indicated the need for additional training were consistently more likely to provide answers indicative of moral distress. These findings may relate to inadequate organisational preparedness and resource availability for staff working in primary, community, and aged care settings. A report prepared by the Aged Care Quality and Safety Commission echoes these concerns and characterised the overwhelming of aged care facilities with one provider stating: ‘We already had a COVID-plan, but we didn’t really prepare for the avalanche of it all’ [29]. Although previous work on predictors of moral distress in HCWs is scarce, people with prior mental health diagnoses are frequently vulnerable to increased psychosocial harm during COVID-19, and it is unsurprising this extends to moral distress [30,31]. Those living and working in metropolitan areas may have been at increased risk of moral distress due to the greater concentration of COVID-19 cases in metropolitan areas. Therefore, these participants had greater exposure to the issues posed in the moral distress questions. At the closure of the study, Australia had recorded 27,484 cases of COVID-19, of which 20,330 were located in Victoria, with most in metropolitan Melbourne [20]. Despite high caseloads, residing in Victoria only increased the likelihood of endorsing concerns about PPE usage limiting ability to care. This may be reflective of the prolonged usage of PPE in routine care settings in Victoria. HCWs in Victoria may have also become more accustomed to enforcing visiting restrictions and managing furloughed staff and resource shortages to the extent where they did not experience additional moral distress on these indicators compared to their interstate counterparts.

Nurses and allied health workers relative to medical staff and ICU staff and other frontline areas relative to ED workers were frequently identified as having reduced odds of reporting moral distress. These results are somewhat surprising given professional autonomy is a frequent predictor of moral distress [32,33] and is generally greater for medical staff than nurses [34,35]. It is possible that greater decisional authority was available for nursing and allied health staff in the context of COVID-19 in Australia, though this is outside the scope of the current survey and warrants further investigation. ICU workers are uniquely positioned amongst frontline HCWs due to their work setting requiring greater baseline familiarity and confidence in working under strict PPE guidelines, which may have protected them from additional moral distress during this time. It is somewhat unexpected that ICU bed shortages witnessed in international settings, and at times, predicted in Australia, did not result in additional moral distress for Australian ICU workers. This may be indicative of successful preparative measures undertaken by these departments in combination with relatively low hospitalisation rates of COVID-19 patients in Australia. The effect of receiving PPE training as well as working with COVID-19 patients on moral distress also concur with recent studies that have demonstrated positive correlations between the scarcity of critical resources, excessive workload, and moral distress during the pandemic [36,37].

In contrast to some prior studies of baseline moral distress in Iranian [38], Canadian [39], and American [40] HCW cohorts, gender was not a frequent predictor variable in the current study. Similarly, younger age was not an independent predictor of moral distress despite being identified in baseline studies of HCWs in Saudi Arabia [41] and Iran [38]. People in age groups over 30 were, however, more likely to endorse statements about the community fearing spread of the virus by HCWs. Prior evidence of impacts of age and gender on experiences of moral distress are mixed and likely confounded by other variables, including education or experience level and professional autonomy. Notably, the current study was sufficiently powered for multiple regression allowing delineation of these confounding variables, which was not possible in prior studies of similar cohorts.

### 4.2. Moral Distress Is Associated with Adverse Mental Health Outcomes

Endorsing indicators of moral distress was frequently found to be independently associated with moderate to severe symptoms of mental illness on all the validated mental health outcomes tested in the current study. Moral distress has been linked to increased turnover within organisations and attrition of HCWs [42,43]; failure to address moral distress early may exacerbate staff burnout, which presents possible risks to patients and co-workers. Of the indicators listed, ‘wearing PPE limiting the ability to care for patients’ and ‘being required to quarantine lets down co-workers’ were the most frequently associated with adverse mental health outcomes. Limitations on ability to provide adequate care is likely reflective of harms associated with a lack of professional autonomy, wherein HCWs are at a greater risk of psychosocial distress when denied the ability to advocate for their patients [34] and operate in accordance with their expertise [44]. Fears of ‘letting down’ co-workers potentially relate to both the stigma associated with contracting COVID-19 and a desire to shelter co-workers from the known risks associated with excessive workloads [45]. Perceived stigma has previously been shown to be a barrier for COVID-19 testing in young or culturally diverse communities in Australia [46]. These results indicate a need for organisational support for HCWs to destigmatise infection with COVID-19, as well as reinforcing surge workforce capacity for future crises.

The demands placed upon HCWs during a time of crises can instil in some workers a sense of meaning or purpose. Qualitative interviews with HCWs in the aftermath of the Haiyan typhoon in the Philippines identified altruistic motives as a means of finding acceptance and control in their circumstances [47]. In the context of COVID-19, a survey of 657 HCWs during the peak of inpatient admissions in New York city reported 61% of participants as feeling an increased sense of meaning [48]. In the current study, participants who agreed that the community was appreciative of HCWs during COVID-19 were less likely to show moderate to severe symptoms of all mental illnesses tested. This may be indicative of a similar positive reframing mindset in which HCWs are able to find altruistic purpose in their work, which in turn, buffers their mental health and provides validation for public and private initiatives to thank or reward HCWs during COVID-19.

### 4.3. Solutions and Interventions to Manage Moral Distress

Our findings reveal that HCWs in certain frontline areas, metropolitan locations, and those with prior mental health diagnoses were disproportionately impacted by moral distress during COVID-19. The causes for these disproportionate impacts are likely multifactorial and relate to resourcing shortages, concentration of caseloads, and known vulnerable demographic factors. Although some of these factors, such as case distribution, are uncontrollable, these results are indicative of where targeted efforts can be made to mitigate moral distress and associated adverse mental health outcomes.

Due to the pandemic, many hospitals and healthcare organisations set up wellbeing supports, which were previously either not available or severely limited [49]. Given the protective effects of perceived community appreciation on mental health, building resilience in the form of positive reframing may be beneficial. Although the causes for reduced moral distress observed in ICU workers cannot be fully elucidated, it is possible that greater baseline familiarity with strict PPE protocols and resource management were partially protective. Whilst the sudden onset of the pandemic has necessitated broadly reactive, rather than proactive, training opportunities for PPE usage, there is an argument for broader PPE and resource management competency training in other frontline areas in preparation for ongoing and future crisis situations. Evidence-based policy development encompassing whole-of-organisation approaches as well as initiatives to reframe these psychological stresses as organisational and collective phenomenon are essential in navigating moral distress [23,50,51].

### 4.4. Strengths, Limitations, and Future Directions

The survey included participants from a wide range of healthcare professions and represents the experiences of HCWs across different frontline specialities. The majority of participants in the current study were women, which is consistent with data from both the Australian Institute of Health and Welfare and the Australian Health Practitioner Regulation Agency demonstrating that 75% of the Australian health workforce is female [52,53]. Due to the broad survey dissemination strategy, calculation of a response rate was not possible, and selection or response bias may have led to over- or under-estimation of moral distress and adverse mental health outcomes.

Due to the spontaneous and unexpected nature of the COVID-19 pandemic, no baseline data regarding moral distress in non-pandemic times had previously been collected from a large cohort of Australian healthcare workers. The design of the survey as a single point-in-time data collection was chosen to minimise burden on HCWs; however, future research is required to provide longitudinal follow-up. In the interests of brevity and to focus on COVID-19, we generated questions relating to specific factors of moral distress. These questions targeted similar dimensions of moral distress to those included in longer, validated scales such as the Measure of Moral Distress for Healthcare Professionals (MMD-HP), such as exclusion of family, acting in accordance with personal values as a HCW, and resource scarcity negatively impacting patient care [54]. Additionally, factors specifically related to the COVID-19 pandemic (such as stigma or appreciation from the community and fear of letting co-workers down if infected with COVID-19) were included. Although this snapshot of factors related to moral distress in the broader context of COVID-19 provides valuable insight, further research in this diverse cohort is required and would benefit from the use of a comprehensive validated scale. This would enable attention to the long-term implications of moral distress and mental health outcomes of this population.

## 5. Conclusions

This large-scale survey provided an insight into predictors of moral distress and its correlation with mental health outcomes in HCWs. Given that a healthy workforce is pivotal to effective healthcare service delivery, recognising and identifying moral distress and its downstream effects as well as promoting the development of targeted interventions and evidence-based policies will contribute to the cultivation of moral resilience in HCWs at workplace and community settings.

## Figures and Tables

**Table 1:** Participants’ characteristics.

Characteristic	Frequency (*n* = 7846)	%
**Age (years)**		
20–30	1860	23.7
31–40	2250	28.7
41–50	1738	22.2
>50	1998	25.5
Gender		
Male	1458	18.6
Female	6344	80.9
Non-binary	19	0.2
Prefer not to say	25	0.3
**Number of years working since graduating (*n* = 6637)**		
0–5	1592	24.0
6–10	1377	20.7
11–15	943	14.2
≥ 15	2725	41.1
**Number of people in the household**		
Lives alone (1 person)	1087	13.9
2	2492	31.8
3–4	3181	40.5
5–6	1024	13.1
≥ 7	62	0.8
**Number of children < 16 years at home**		
0	5102	65.0
1–2	2253	28.7
3–4	482	6.1
≥ 5	9	0.1
**Lives with ≥ 1 elderly person/people at home**	697	8.9

**Table 2:** Moral distress and perceived community views of healthcare workers.

Characteristic	Frequency	%
**Indicators of Moral Distress**		
Worried that some patients will not receive the care they need due to scarcity of resources		
Strongly agree	1582	20.2
Somewhat agree	2986	38.1
Neither agree not disagree	1211	15.4
Somewhat disagree	1299	16.6
Strongly disagree	768	9.8
Wearing PPE means that they cannot properly provide care to people with COVID-19		
Strongly agree	702	8.9
Somewhat agree	2027	25.8
Neither agree not disagree	1861	23.7
Somewhat disagree	1671	21.3
Strongly disagree	1585	20.2
Being required to quarantine lets down co-workers who are already overworked and stressed		
Strongly agree	1643	20.9
Somewhat agree	2675	34.1
Neither agree not disagree	1203	15.3
Somewhat disagree	1128	14.4
Strongly disagree	1197	15.3
Excluding family from the bedside of patients infected with COVID-19 goes against their values as a healthcare worker		
Strongly agree	2000	25.5
Somewhat agree	2720	34.7
Neither agree not disagree	1607	20.5
Somewhat disagree	951	12.1
Strongly disagree	568	7.2
**Perceived Attitudes to Healthcare Workers’**		
The community is worried that healthcare workers spread the virus to others		
Neither agree nor disagree	1052	13.4
Strongly/somewhat disagree	777	9.9
Strongly/somewhat agree	6017	76.7
The community is appreciative of healthcare workers during this time		
Neither agree nor disagree	665	8.5
Strongly/somewhat disagree	397	5.1
Strongly/somewhat agree	6784	86.5

**Table 3:** Personal and workplace predictors of moral distress and perceived community view (multivariate analysis).

	Moral Distress								Perceived Community Views			
	Worried That Some Patients Will Not Receive the Care They Need Due to Scarcity of Resources		Wearing PPE Means That They Cannot Properly Provide Care to People with COVID-19		Being Required to Quarantine Lets Down Co-Workers Who Are Already Overworked and Stressed		Excluding Family from the Bedside of Patients Infected with COVID-19 Goes against Their Values as a Healthcare Worker		the Community Is Worried That Healthcare Workers Spread the Virus to Others		the Community Is Appreciative of Healthcare Workers during This Time	
	OR (95% CI)	*p*	OR (95% CI)	*p*	OR (95% CI)	*p*	OR (95% CI)	*p*	OR (95% CI)	*p*	OR (95% CI)	*p*
**Personal Predictors**												
**Age (y)**												
31–40	1.23 (0.91–1.64)	0.176	0.85 (0.64–1.14)	0.281	1.21 (0.91–1.62)	0.186	0.92 (0.71–1.20)	0.559	2.20 (1.56–3.09)	0.001	0.57 (0.37–0.88)	0.010
41–50	1.11 (0.87–1.43)	0.397	0.81 (0.63–1.03)	0.088	1.11 (0.87–1.42)	0.386	0.84 (0.67–1.05)	0.133	1.46 (1.10–1.93)	0.008	0.69 (0.47–1.02)	0.062
50 +	0.91 (0.75–1.10)	0.329	0.92 (0.77–1.12)	1.411	0.96 (0.79–1.16)	0.672	0.95 (0.79–1.13)	0.550	1.26 (1.01–1.57)	0.037	0.74 (0.54–1.02)	0.069
**Gender**	N/A	-	N/A	-	N/A	-	2.01 (1.74–2.33)	0.001	N/A	-	N/A	-
**State (VIC)**	0.85 (0.69–1.06)	0.144	1.42 (1.14–1.76)	0.002	0.84 (0.69–1.04)	0.103	N/A	-	1.01 (0.80–1.29)	0.907	N/A	-
**Pre-existing mental health condition**	1.26 (1.09–1.45)	0.002	N/A	-	1.25 (1.09–1.44)	0.002	1.29 (1.14–1.47)	0.001	N/A	-	1.01 (0.82–1.24)	0.928
**Workplace Predictors**												
**Occupation**												
Nursing	0.58 (0.49–0.68)	0.001	0.66 (0.57–0.77)	0.001	0.58 (0.49–0.67)	0.001	0.64 (0.56–0.74)	0.001	1.07 (0.89–1.29)	0.461	0.62 (0.50–0.78)	0.001
Allied health	0.78 (0.62–0.98)	0.031	0.73 (0.58–0.91)	0.005	0.80 (0.64–1.00)	0.048	0.80 (0.65–0.97)	0.023	0.88 (0.68–1.14)	0.316	0.89 (0.64–1.24)	0.496
Other role	0.68 (0.46–1.02)	0.063	0.42 (0.28–0.66)	0.001	0.70 (0.48–1.02)	0.063	0.52 (0.38–0.73)	0.001	0.53 (0.36–0.80)	0.002	1.57 (1.07–2.29)	0.020
**Frontline Area**												
ICU	0.66 (0.53–0.83)	0.001	0.84 (0.67–1.04)	0.116	0.64 (0.51–0.79)	0.001	0.76 (0.62–0.93)	0.009	1.18 (0.90–1.55)	0.226	N/A	-
Anaesthetics and surgery	1.02 (0.80–1.31)	0.848	0.82 (0.64–1.04)	0.106	1.01 (0.80–1.27)	0.944	0.54 (0.44–0.66)	0.001	0.81 (0.61–1.24)	0.122	N/A	-
Medical specialty areas	1.07 (0.88–1.29)	0.503	1.02 (0.84–1.22)	0.877	1.05 (0.88–1.25)	0.611	0.81 (0.69–0.96)	0.012	1.12 (0.90–1.40)	0.322	N/A	-
Primary care, community and aged care	1.52 (1.14–2.04)	0.005	1.00 (0.76–1.34)	0.976	1.46 (1.11–1.91)	0.007	0.87 (0.68–1.10)	0.239	1.10 (0.80–1.55)	0.539	N/A	-
Other frontline area *	0.84 (0.61–1.14)	0.259	0.84 (0.60–1.15)	0.272	0.85 (0.63–1.15)	0.294	0.62 (0.47–0.82)	0.001	0.87 (0.61–1.24)	0.447	N/A	-
**Works in a metropolitan area**	1.28 (1.06–1.56)	0.012	0.99 (0.82–1.20)	0.922	1.23 (1.02–1.48)	0.027	1.17 (1.00–1.38)	0.056	0.96 (0.77–1.18)	0.672	N/A	-
**Currently works with COVID-19 patients**	1.01 (0.86–1.18)	0.932	1.44 (1.23–1.68)	0.001	N/A	-	N/A	-	1.02 (0.85–1.22)	0.830	0.76 (0.62–0.93)	0.009
**Received PPE training**	1.00 (0.80–1.24)	0.978	1.24 (0.99–1.54)	0.058	N/A	-	N/A	-	0.99 (0.78–1.27)	0.959	1.40 (1.06–1.86)	0.020
**Confident using PPE**	1.79 (0.62–1.01)	0.063	0.76 (0.60–0.96)	0.020	N/A	-	N/A	-	N/A	-	1.44 (1.05–1.96)	0.023
**Desires more training regarding PPE or managing COVID-19**	1.66 (1.44–1.90)	0.001	1.25 (1.08–1.43)	0.002	1.68 (1.47–1.91)	0.001	1.15 (1.03–1.30)	0.016	N/A	-	0.92 (0.75–1.13)	0.443

* Other frontline area = people working in paramedicine, radiology, pharmacy, pathology, maintenance, administrative staff, and COVID-19 screening. N/A = variable not included for that outcome question in the model as there was no relationship seen in the univariate model. OR = odds ratio. 95% CI = 95% confidence interval. Baseline reference categories for each variable were: age = 20–30 years; gender = male; state = other states; pre-existing mental health conditions = negative response; occupation = medical staff; frontline area = people working in ED; work location = regional or remote area; currently works with COVID-19 patients = negative response; received PPE training = negative response; confidence using PPE = negative response; needs more training using PPE and managing COVID-19 patients = negative response.

**Table 4:** Relationship between moral distress and mental health outcomes (multivariate analysis).

Characteristics	GAD-7		PHQ-9		IES-6		Burnout-DP		Burnout-EE		Burnout-PA	
	OR (95% CI)	*p*	OR (95% CI)	*p*	OR (95% CI)	*p*	OR (95% CI)	*p*	OR (95% CI)	*p*	OR (95% CI)	*p*
**Predictors**												
Worried that some patients will not receive the care they need due to resource scarcity	1.09 (0.98–1.21)	0.136	0.97 (0.87–1.09)	0.647	1.15 (1.05–1.27)	0.005	1.17 (1.06–1.29)	0.002	1.18 (1.07–1.31)	0.002	1.17 (1.06–1.30)	0.002
Wearing PPE means that they cannot properly provide care to people with COVID-19	1.28 (1.14–1.42)	<0.0001	1.13 (1.01–1.27)	0.031	1.30 (1.17–1.44)	<0.0001	1.48 (1.33–1.63)	<0.0001	1.28 (1.14–1.43)	<0.0001	N/A	-
Being required to quarantine lets down co-workers who are already overworked and stressed	2.00 (1.79–2.23)	<0.0001	1.73 (1.55–1.94)	<0.0001	1.91 (1.73–2.10)	<0.0001	1.29 (1.17–1.42)	<0.0001	1.79 (1.61–1.99)	<0.0001	N/A	-
Excluding family from the bedside goes against their values	1.15 (1.03–1.28)	0.014	1.18 (1.05–1.33)	0.004	1.23 (1.11–1.36)	<0.0001	N/A	-	1.13 (1.02–1.26)	0.019	1.30 (1.18–1.44)	<0.0001
The community is worried that HCWs spread the virus to others	1.28 (1.13–1.46)	<0.0001	1.12 (0.99–1.28)	0.081	1.50 (1.33–1.70)	<0.0001	1.37 (1.22–1.53)	<0.0001	1.49 (1.33–1.67)	<0.0001	N/A	-
The community is appreciative of HCWs during this time	0.44 (0.38–0.50)	<0.0001	0.60 (0.52–0.70)	<0.0001	0.53 (0.46–0.61)	<0.0001	0.59 (0.52–0.70)	<0.0001	0.58 (0.49–0.68)	<0.0001	1.62 (1.41–1.86)	<0.0001

N/A = variable not included for that mental scale in the model as there was no relationship seen in the univariate model. Reference category for each variable were: worried about scarcity of resources = negative response; wearing PPE limits proper care for COVID-19 patients = negative response; quarantine lets down workers = negative response; excluding family visits to COVID-19 patients goes against HCW values = negative response; community worries HCW spread the virus = negative response; community appreciates HCW during the pandemic = negative response. GAD-7 = Generalized Anxiety Disorder Scale; PHQ-9 = Patient Health Questionnaire; IES-6 = abbreviated Impact of Event Scale; Burnout DP = depersonalisations; EE = emotional exhaustion; PA = personal accomplishment; OR = odds ratio.

## Data Availability

Data available upon reasonable request to corresponding author.

## Supplementary Materials

The following are available online at https://www.mdpi.com/article/10.3390/ijerph18168723/s1, Survey questionnaire.

## References

[1] Weingarten K., Galvan-Duran A.R., D’Urso S., Garcia D. (2020). The Witness to Witness Program: Helping the Helpers in the Context of the COVID-19 Pandemic. Fam. Process.

[2] Lesley M. (2021). Psychoanalytic Perspectives on Moral Injury in Nurses on the Frontlines of the COVID-19 Pandemic. J. Am. Psychiatr. Nurses Assoc..

[3] Litz B.T., Stein N., Delaney E., Lebowitz L., Nash W.P., Silva C. (2009). Moral injury and moral repair in war veterans: A preliminary model and intervention strategy. Clin. Psychol. Rev..

[4] Williamson V., Stevelink S.A.M., Greenberg N. (2018). Occupational moral injury and mental health: Systematic review and meta-analysis. Br. J. Psychiatry.

[5] Rice E.M., Rady M.Y., Hamrick A., Verheijde J.L., Pendergast D.K. (2008). Determinants of moral distress in medical and surgical nurses at an adult acute tertiary care hospital. J. Nurs. Manag..

[6] Hines S.E., Chin K.H., Glick D.R. (2021). Trends in Moral Injury, Distress, and Resilience Factors among Healthcare Workers at the Beginning of the COVID-19 Pandemic. Int. J. Environ. Res. Public Health.

[7] Nickell L.A., Crighton E.J., Tracy C.S., Al-Enazy H., Bolaji Y., Hanjrah S. (2004). Psychosocial effects of SARS on hospital staff: Survey of a large tertiary care institution. Can. Med. Assoc. J..

[8] El Gaafary M.M., Abd Elaziz K.M., Abdel-Rahman A.G., Allam M.F. (2010). Concerns, perceived impacts and preparedness of health care workers in a referral hospital in Egypt in facing influenza (H1N1) epidemic. J. Prev. Med. Hyg..

[9] Witter J.R.H.W.S. (2018). Health workers’ experiences of coping with the Ebola epidemic in Sierra Leone’s health system: A qualitative study. BMC Health Serv. Res..

[10] Rimmer A. (2020). Covid-19: Two fifths of doctors say pandemic has worsened their mental health. BMJ.

[11] (2019). Beyond, Blue, National Mental Health Survey of Doctors and Medical Students, Beyond Blue, Editor. https://www.beyondblue.org.au/docs/default-source/research-project-files/bl1132-report---nmhdmss-full-report_web.

[12] Hu D., Kong Y., Li W., Han Q., Zhang X., Zhu L.X. (2020). Frontline nurses’ burnout, anxiety, depression, and fear statuses and their associated factors during the COVID-19 outbreak in Wuhan, China: A large-scale cross-sectional study. EClinicalMedicine.

[13] Pappa S., Ntella V., Giannakas T., Giannakoulis V.G., Papoutsi E., Katsaounou P. (2020). Prevalence of depression, anxiety, and insomnia among healthcare workers during the COVID-19 pandemic: A systematic review and meta-analysis. Brain Behav. Immun..

[14] Dobson H., Malpas C.B., Burrell A.J., Gurvich C., Chen L., Kulkarni J. (2021). Burnout and psychological distress amongst Australian healthcare workers during the COVID-19 pandemic. Australas. Psychiatry.

[15] Tawfik D.S., Scheid A., Profit J., Shanafelt T., Trockel M., Adair K.C. (2019). Evidence Relating Health Care Provider Burnout and Quality of Care: A Systematic Review and Meta-analysis. Ann. Intern. Med..

[16] Shanafelt T.D., Boone S., Tan L., Dyrbye L.N., Sotile W., Satele D. (2012). Burnout and satisfaction with work-life balance among US physicians relative to the general US population. Arch. Intern. Med..

[17] De Kock J.H., Latham H.A., Leslie S.J., Grindle M., Munoz S.A., Ellis L. (2021). A rapid review of the impact of COVID-19 on the mental health of healthcare workers: Implications for supporting psychological well-being. BMC Public Health.

[18] Ghasemi E., Negarandeh R., Janani L. (2019). Moral distress in Iranian pediatric nurses. Nurs. Ethics.

[19] Austin C.L., Saylor R., Finley P.J. (2017). Moral distress in physicians and nurses: Impact on professional quality of life and turnover. Psychol. Trauma.

[20] (2020). Coronavirus (COVID-19) at a Glance—23 October 2020. https://www.health.gov.au/resources/publications/coronavirus-covid-19-at-a-glance-23-october-2020.

[21] Harris P.A., Taylor R., Thielke R., Payne J., Gonzalez N., Conde J.G. (2009). Research electronic data capture (REDCap)—A metadata-driven methodology and workflow process for providing translational research informatics support. J. Biomed. Inform..

[22] Gustavsson M.E., Arnberg F.K., Juth N., von Schreeb J. (2020). Moral Distress among Disaster Responders: What is it?. Prehosp. Disaster Med..

[23] A New Guide to Managing Moral Injury in Healthcare Workers during COVID-19. https://www.phoenixaustralia.org/moral-injury-in-healthcare-workers-during-covid-19/.

[24] R Studio Team (2015). RStudio: Integrated Development Environment for R. http://www.rstudio.com/.

[25] Riley M.R., Mohr D.C., Waddimba A.C. (2018). The reliability and validity of three-item screening measures for burnout: Evidence from group-employed health care practitioners in upstate New York. Stress Health.

[26] Thoresen S., Tambs K., Hussain A., Heir T., Johansen V.A., Bisson J.I. (2009). Brief measure of posttraumatic stress reactions: Impact of Event Scale-6. Soc. Psychiatry Psychiatr. Epidemiol..

[27] Spitzer R.L., Kroenke K., Williams J.B.W., Löwe B. (2006). A Brief Measure for Assessing Generalized Anxiety Disorder: The GAD-7. Arch. Intern. Med..

[28] Kroenke K., Spitzer R.L., Williams J.B. (2001). The PHQ-9: Validity of a brief depression severity measure. J. Gen. Intern. Med..

[29] (2020). “We Saw the Best in People” Lessons Learned by Aged Care Providers Experiencing Outbreaks of COVID-19 in Victoria, Australia. https://www.agedcarequality.gov.au/media/89099.

[30] Fancourt D., Steptoe A., Bu F. (2020). Trajectories of depression and anxiety during enforced isolation due to COVID-19: Longitudinal analyses of 36,520 adults in England. medRxiv.

[31] Bu F., Steptoe A., Fancourt D. (2020). Loneliness during a strict lockdown: Trajectories and predictors during the COVID-19 pandemic in 38,217 United Kingdom adults. Soc. Sci. Med..

[32] Abdolmaleki M., Lakdizaji S., Ghahramanian A., Allahbakhshian A., Behshid M. (2019). Relationship between autonomy and moral distress in emergency nurses. Indian J. Med. Ethics.

[33] Dodek P., Norena M., Ayas N., Dhingra V., Brown G., Wong H. (2019). Moral distress in intensive care unit personnel is not consistently associated with adverse medication events and other adverse events. J. Crit. Care.

[34] Bender M.A., Andrilla C.H.A., Sharma R.K., Hurd C., Solvang N., Mae-Baldwin L. (2019). Moral Distress and Attitudes about Timing Related to Comfort Care for Hospitalized Patients: A Survey of Inpatient Providers and Nurses. Am. J. Hosp. Palliat. Care.

[35] Fruet I.M.A., Dalmolin G.d.L., Bresolin J.Z., Andolhe R., Barlem E.L.D. (2019). Moral Distress Assessment in the Nursing Team of a Hematology-Oncology Sector. Rev. Bras. Enferm..

[36] O’Neal L., Heisler M., Mishori R., Haar R.J. (2021). Protecting providers and patients: Results of an Internet survey of health care workers’ risk perceptions and ethical concerns during the COVID-19 pandemic. Int. J. Emerg. Med..

[37] Cawcutt K.A., Starlin R., Rupp M.E. (2020). Fighting fear in healthcare workers during the COVID-19 pandemic. Infect. Control. Hosp. Epidemiol..

[38] Soleimani M.A., Sharif S.P., Yaghoobzadeh A., Sheikhi M.R., Panarello B., Win M.T.M. (2019). Spiritual well-being and moral distress among Iranian nurses. Nurs. Ethics.

[39] Burns K.E.A., Fox-Robichaud A., Lorens E., Martin C.M. (2019). Gender differences in career satisfaction, moral distress, and incivility: A national, cross-sectional survey of Canadian critical care physicians. Can. J. Anaesth..

[40] O’Connell C.B. (2015). Gender and the experience of moral distress in critical care nurses. Nurs. Ethics.

[41] Almutairi A.F., Salam M., Adlan A.A., Alturki A.S. (2019). Prevalence of severe moral distress among healthcare providers in Saudi Arabia. Psychol. Res. Behav. Manag..

[42] Sajjadi S., Norena M., Wong H., Dodek P. (2017). Moral distress and burnout in internal medicine residents. Can. Med. Educ. J..

[43] Whitehead P.B., Herbertson R.K., Hamric A.B., Epstein E.G., Fisher J.M. (2015). Moral distress among healthcare professionals: Report of an institution-wide survey. J. Nurs. Sch..

[44] Doobay-Persaud A., Evert J., DeCamp M., Evans C.T., Jacobsen K.H., Sheneman N.E., Goldstein J.L., Nelson B.D. (2019). Extent, nature and consequences of performing outside scope of training in global health. Glob. Health.

[45] Petrie K., Crawford J., LaMontagne A.D., Milner A., Dean J., Veness B.G., Christensen H., Harvey S.B. (2020). Working hours, common mental disorder and suicidal ideation among junior doctors in Australia: A cross-sectional survey. BMJ Open.

[46] Shame and Stigma Barriers to COVID-19 Testing for Young Australians and Culturally Diverse Communities, New Research Finds. https://burnet.edu.au/system/asset/file/4601/Optimise_Media_Release_-_Shame_and_stigma_barriers_to_COVID-19_testing_-_FINAL.pdf.

[47] Hugelius K., Adolfsson A., Ortenwall P., Gifford M. (2017). Being Both Helpers and Victims: Health Professionals’ Experiences of Working during a Natural Disaster. Prehosp. Disaster Med..

[48] Shechter A., Diaz F., Moise N., Anstey D.E., Ye S., Agarwal S., Birk J.L., Brodie D., Cannone D.E., Chang B. (2020). Psychological distress, coping behaviors, and preferences for support among New York healthcare workers during the COVID-19 pandemic. Gen. Hosp. Psychiatry.

[49] Mellins C.A., Mayer L.E.S., Glasofer D.R., Devlin M.J., Albano A.M., Nash S.S., Engle E., Cullen C., Ng W.Y.K., Allmann A.E. (2020). Supporting the well-being of health care providers during the COVID-19 pandemic: The CopeColumbia response. Gen. Hosp. Psychiatry.

[50] Nguyen Q.K.J., Naswall K., Malinen S. (2016). Employee resilience and leadership styles: The moderating role of proactive personality and optimism. N. Z. J. Psychol..

[51] Forstag E.H., Cuff P.A. (2018). A Design Thinking, Systems Approach to Well-Being Within Education and Practice: Proceedings of a Workshop.

[52] Medical Board of Australia (2020). Registration Data Table—December 2020.

[53] Nursing and Midwifery Board (2020). Nursing and Midwifery Board of Australia Registrant Data.

[54] Epstein E.G., Whitehead P.B., Prompahakul C., Thacker L.R., Hamric A.B. (2019). Enhancing Understanding of Moral Distress: The Measure of Moral Distress for Health Care Professionals. AJOB Empir. Bioeth..

