# Supplementary material for: Moral Distress and Perceived Community Views Are Associated with Mental Health Symptoms in Frontline Health Workers during the COVID-19 Pandemic
Source: Int J Environ Res Public Health. 2021 Aug 18;18(16):8723. doi: 10.3390/ijerph18168723 (PMC8392524; doi:10.3390/ijerph18168723)
Supplement: Supplementary file 1 [file ijerph-18-08723-s001.zip › ijerph-1334156-supplementary.pdf]

Future Proofing Frontline Healthcare Workers in Times of Pandemic and Other Crises

Explanatory Statement

You are invited to take part in this study. Please read this statement before deciding whether or not to participate in this research. If you would like further information regarding any aspect of this project, you are encouraged to visit our website: https://www.covid-19-frontline.com.au

What does the research involve?

The aim of the study is to explore the social, work and mental health effects experienced by frontline health workers during the COVID-19 pandemic and beyond. We aim to examine factors that promote good mental health and risk factors that contribute to poorer mental health.

Participating in this study will involve completing an online questionnaire, which has been piloted and shown to take approximately 15-20 minutes. These questions will include information about you and your work, what you do to stay healthy, and the impact the COVID-19 pandemic has had on your health and wellbeing, your social situation, and your work.

Why were you chosen for this research?

You have been invited to participate in this study because you are a ‘frontline health worker’. In this study fontline health workers are defined as medical, nursing, or allied health professionals, clinical scientists/physiologists/technicians, healthcare students or clerical staff working in hospitals, primary care, the community, private practice or paramedicine.

You have been invited via your Head of Department/Director of Training/manager, your professional association, a colleague or friend, or by advertisement of this study.

You do NOT need to have worked with patients with COVID-19 to take part. We would like to hear from both people who have and who have not worked with people with COVID-19.

Consenting to participate in this project and withdrawing from the research

Participation in this study is voluntary. It is your decision whether to participate. If at any stage, you feel uncomfortable or you feel the questions are too personal, you may stop completing the survey and close it. As this study is an anonymous survey once you submit any answers withdrawal of data will not be possible.

Possible benefits and risks to participate

There are unlikely to be any immediate direct benefits from participating in the study, other than reflecting on your current mental health and wellbeing. Participating in this study will provide data that will help us understand the supports that frontline healthcare workers need and want when confronted with a crisis situation like COVID-19. This information is important for supporting health workers during futures crises.

We do not anticipate that there are immediate risks to you; but we acknowledge that reflecting on the impact of COVID-19 may arouse feelings of disquiet or distress. If this is the case, we encourage you to seek support and we have provided advice and links on our website and at the conclusion of the survey.

Confidentiality

The survey is anonymous and confidential. At no point in the study will we be asking your name, date of birth or address. Your survey results will not be considered individually, instead the results from all the participants will be combined for analysis to provide overall trends when data are presented

Storage of Data

Data will be collected through a Melbourne Health account on REDcap, which is password protected. 2 It will only be accessible by the researchers involved in the study. Data will be kept for 5 years and then destroyed via deletion of electronic files when no longer needed.

Results

Once the study is complete, a summary of the findings will be posted on our website. Interim analyses (after 2 months) of our research will also be made available on the study website.

Complaints

Should you have any concerns or complaints about the conduct of the project, you are welcome to contact the Manager, Melbourne Health Human Research Ethics Committee, Ph: 03 93428530; or email: research@mh.org.au

---

CONSENT

☐ I have read the explanatory statement. I am a health care worker. I am over 18 years of age and I agree to participate.

PART A - Demographics and Home Life

Survey Progress

How old are you?	◯ 20-30<br>◯ 31-40<br>◯ 41-50<br>◯ 50-64<br>◯ 65-70<br>◯ 71+
What is your gender?	◯ Male<br>◯ Female<br>◯ Non-binary<br>◯ Prefer not to say
How many people, including yourself live in your household?	__________________
How many people aged 65 years or older live in your house?	__________________
How many children (under 16 years) live in your house?	__________________
Since the COVID-19 pandemic started, have you had to actively manage home schooling responsibilities?	◯ Yes<br>◯ No<br>◯ Not Applicable
Do you have any caring responsibilities (such as for children, older adults or others) that have impacted your ability to work during the pandemic?	◯ Yes<br>◯ No<br>◯ Not Applicable

PART B - Professional Background and Work Arrangements

Survey Progress

Which state do you work in?	◯ South Australia<br>◯ Victoria<br>◯ ACT<br>◯ New South Wales<br>◯ Northern Territory<br>◯ Queensland<br>◯ Tasmania<br>◯ Western Australia
Please tick your places of work (select all that apply)?	☐ Public hospital<br>☐ Private hospital<br>☐ Other<br>☐ Community
If other, please specify	__________________________________
Where is the main location of your clinical work?	◯ Metropolitan area<br>◯ Regional area<br>◯ Remote area
What is your profession or work background?	◯ Senior Medical Staff<br>◯ General Practitioner<br>◯ Junior Medical Staff<br>◯ Nursing<br>◯ Allied Health<br>◯ Clinical Scientist/Physiologist/Technician<br>◯ Student - Medical, Nursing, Allied Health<br>◯ Clerical or Administrative Staff<br>◯ Paramedic<br>◯ Other
Please specify Allied Health profession	◯ Physiotherapist<br>◯ Occupational Therapist<br>◯ Speech Pathologist<br>◯ Social Worker<br>◯ Psychologist<br>◯ Technician<br>◯ Medical Scientist<br>◯ Other
If other, please specify	__________________________________
If other, please specify	__________________________________
How many years have your worked in your profession since graduation?	◯ 0-5 years<br>◯ 6-10 years<br>◯ 11-15 years<br>◯ More than 15 years

How many years have your worked in your current role?	◯ 0-5 years<br>◯ 6-10 years<br>◯ 11-15 years<br>◯ More than 15 years
What health course are you studying?	◯ Allied Health<br>◯ Applied Medical Science<br>◯ Medicine<br>◯ Nursing<br>◯ Paramedicine
As a student what year are you in for your healthcare course?	◯ 1st Year<br>◯ 2nd Year<br>◯ 3rd Year<br>◯ 4th Year or more
Which frontline area do you work with? If you work on an inpatient ward, please select the department below that is most associated with your ward.	◯ Emergency Department<br>◯ Intensive Care Unit<br>◯ Respiratory Medicine<br>◯ General Medicine<br>◯ Infectious Diseases<br>◯ Other Medical Specialty Area<br>◯ Surgical Specialty Area<br>◯ Anaesthetics /Perioperative Care<br>◯ Hospital Aged Care<br>◯ Palliative Care<br>◯ Primary Care or Community<br>◯ Paramedic<br>◯ Other
If other, please specify	__________________________________
Do you CURRENTLY work in direct contact with COVID-19 patients?	◯ Yes<br>◯ No
Do you ANTICIPATE that you may have to work in direct contact with COVID-19 patients if numbers increase in your state?	◯ Yes<br>◯ No
Have you received training to care for patients with COVID-19? (please select all that apply)	☐ Yes - regarding caring for patients with COVID-19<br>☐ Yes - regarding the use of personal protective equipment (PPE)<br>☐ Yes - other<br>☐ No
If other, please specify	__________________________________

	very unconfident 1	2	3	neutral 4	5	6	very confident 7	N/A
How confident do you feel to care for patients with COVID-19	◯	◯	◯	◯	◯	◯	◯	◯

How confident do you feel using PPE when coming into contact with patients with COVID-19	◯	◯	◯	◯	◯	◯	◯	◯

Do you feel you need more training related to COVID-19 or using PPE?

◯ Yes
◯ No

	not worried at all 1	2	3	neutral 4	5	6	very worried 7
How worried are you about the possibility of your role leading to transmission of COVID-19 to your family?	◯	◯	◯	◯	◯	◯	◯

PART C - Work and Finances: Impact of COVID-19 Pandemic

Survey Progress

We would like to know if your working arrangements have changed due to the COVID-19 pandemic:

BEFORE the COVID-19 pandemic what was your employment status?

- ◯ Full time
- ◯ Part time
- ◯ Casual
- ◯ Other

If other, please specify

____

CURRENTLY what is your employment status?

- ◯ Full time
- ◯ Part time
- ◯ Casual
- ◯ Other

If other, please specify

____

Have your paid or unpaid hours spent working changed? This includes work carried out in the hospital or from home. Please select all that apply

- ☐ Increased paid hours
- ☐ Increased unpaid hours
- ☐ Decreased hours (paid or unpaid)
- ☐ No change

Have you been redeployed to a new area of work (i.e. change in the department you work in)?

- ◯ Yes
- ◯ No

	very unconfident 1	2	3	neutral 4	5	6	very confident 7
How confident do you feel working in your new area?	◯	◯	◯	◯	◯	◯	◯

Has your role at work changed?

- ◯ Yes
- ◯ No

	very unconfident 1	2	3	neutral 4	5	6	very confident 7
How confident do you feel working in your new role?	◯	◯	◯	◯	◯	◯	◯

Has your household income altered due to COVID-19?

- ◯ Increased
- ◯ Decreased
- ◯ No change

As a result of COVID-19 do you have concerns or worries about your household income?

- ◯ Yes
- ◯ No

PART D - Exposure to COVID-19

Survey Progress

In the last week, please estimate how many patients with CONFIRMED COVID-19 you have encountered	◯ None<br>◯ 1-5<br>◯ 6-10<br>◯ 11-20<br>◯ 21-50<br>◯ 51-100<br>◯ >100<br>◯ Don't know
In the last week, please estimate how many patients with SUSPECTED COVID-19 you have encountered?	◯ None<br>◯ 1-5<br>◯ 6-10<br>◯ 11-20<br>◯ 21-50<br>◯ 51-100<br>◯ >100<br>◯ Don't know
Have you ever been tested for COVID-19?	◯ Yes<br>◯ No
How many COVID-19 tests have you had since the pandemic started?	________________________
Have you ever had a positive test for COVID-19?	◯ Yes<br>◯ No<br>◯ Prefer not to say<br>◯ Result not known yet
Have you ever been quarantined (i.e. furloughed) due to significant exposure to someone with COVID-19?	◯ Yes<br>◯ No
Do you have close friends or relatives (in Australia or overseas) who have contracted COVID-19?	◯ Yes<br>◯ No

Survey Progress

How true do you believe the following statements are:

	Strongly disagree	Somewhat disagree	Neither agree nor disagree	Somewhat agree	Strongly agree
The community is worried that health workers will spread the virus to others	○	○	○	○	○
The community is generally appreciative of health workers during this time	○	○	○	○	○
If I contract COVID-19, colleagues will question whether I took sufficient precautions	○	○	○	○	○

Has the COVID-19 pandemic had an impact on your relationships with family, friends and work colleagues? (please select all that apply)

- ☐ I have a closer or stronger relationship with my partner
- ☐ I have a closer or stronger relationship with my children/parents/family
- ☐ I have a closer or stronger relationship with my friends
- ☐ I have a closer or stronger relationship with my work colleagues
- ☐ I have a worse relationship with my partner
- ☐ I have a worse relationship with my children/parents/family
- ☐ I have a worse relationship with my friends
- ☐ I have a worse relationship with my work colleagues
- ☐ No effect on relationships

PART E - Relaxing and Staying Healthy

Survey Progress

Question	Response
Generally do you consider that your physical health is:	◯ Excellent<br>◯ Good<br>◯ Fair<br>◯ Poor
Do you have underlying health conditions that you believe increase your risk of becoming unwell with COVID-19?	◯ Yes<br>◯ No
Do you use digital apps to track your PHYSICAL health activities (e.g. exercise, diet, other health measures)?	◯ Yes<br>◯ No
PRIOR to the COVID-19 pandemic have you ever been diagnosed with depression, anxiety, or another mental health condition?	◯ Yes<br>◯ No<br>◯ Prefer not to say
SINCE the COVID-19 pandemic started, do you believe that you have experienced any of the following? (please select all that apply)	☐ Anxiety<br>☐ Burn out<br>☐ Depression<br>☐ Post-traumatic stress disorder<br>☐ Other mental health problem<br>☐ None of the above<br>☐ Prefer not to say
Since the COVID-19 pandemic started, what activities have you undertaken to manage any possible MENTAL HEALTH issues such as stress, anxiety or depression? (please select all that apply)	☐ Maintained exercise<br>☐ Increased exercise<br>☐ Yoga, meditation or similar<br>☐ Maintained or increased social interaction with family and friends<br>☐ Used an app (e.g. Smiling mind, Headspace or other)<br>☐ Increased alcohol use<br>☐ Other strategy<br>☐ None of the above
If other, please specify	__________________________________
If you have used or are currently using an App for stress or to support mental health, which app did you use?	__________________________________
Was the App useful?	◯ Yes<br>◯ No<br>◯ Not Applicable
Are you still using the App?	◯ Yes<br>◯ No<br>◯ Not Applicable
Since the COVID-19 pandemic started,have you sought help from any of the following sources for stress, anxiety, depression or another mental health issue? (please select all that apply)	☐ Doctor or psychologist<br>☐ Employee support program at my place of work<br>☐ Professional support program outside of work<br>☐ None of the above<br>☐ Other

Please specify

____

What do you think would help you most in dealing with stress, anxieties, and other mental health issues (including burnout) related to the COVID-19 pandemic?

____

PART F - My healthcare organisation and organisational changes during COVID-19

Survey Progress

Please answer these questions for the healthcare organisation where you mainly work

The communications I have received so far about workplace changes due to COVID-19 have been useful and timely	◯ Strongly agree<br>◯ Somewhat agree<br>◯ Neither agree nor disagree<br>◯ Somewhat disagree<br>◯ Strongly disagree
How well has your healthcare organisation supported your wellbeing and mental health during the COVID-19 pandemic? For example providing new resources e.g. Apps, telephone support lines etc	◯ Very well supported<br>◯ Somewhat supported<br>◯ Neither supported or unsupported<br>◯ Somewhat unsupported<br>◯ Very unsupported
What did you find to be the main challenges that you faced during the COVID-19 pandemic?	__________________________________
What strategies might be helpful to assist frontline healthcare workers during future crisis events like pandemics, disasters etc?	__________________________________

Survey Progress

The following questions ask about the provision of health care during the COVID-19 pandemic:

	Strongly disagree	Somewhat disagree	Neither agree nor disagree	Somewhat agree	Strongly agree
I am worried that some patients will not receive the care they need due to scarcity of resources.	◯	◯	◯	◯	◯
Having to wear PPE means that I cannot properly provide the care to patients with suspected or confirmed COVID-19.	◯	◯	◯	◯	◯
If I have to go into quarantine, I am letting down my co-workers who are already overworked and stressed.	◯	◯	◯	◯	◯
Excluding close family from the bedside of patients with COVID-19 goes against my values as a healthcare worker	◯	◯	◯	◯	◯

PART G - Measuring wellbeing, resilience, and coping

Survey Progress

The next section asks about your feelings, thoughts, and responses to the COVID-19 pandemic using well validated scales.

Survey Progress

The next 2 questions consider resilience (Connor-Davidson Resilience Scale- the CD-RISC-2)

	Not true at all	Rarely true	Sometimes true	Often true	True nearly all the time
I am able to adapt when changes occur	○	○	○	○	○
I tend to bounce back after illness, injury, or other hardships	○	○	○	○	○

Survey Progress

Over the past TWO WEEKS, how often you have been bothered by the following? (Generalized Anxiety Disorder - GAD-7)

	Not at all	Several days	More than half the days	Nearly every day
Feeling nervous, anxious, or on edge	◯	◯	◯	◯
Not being able to stop or control worrying	◯	◯	◯	◯
Worrying too much about different things	◯	◯	◯	◯
Trouble relaxing	◯	◯	◯	◯
Being so restless that it is hard to sit still	◯	◯	◯	◯
Becoming easily annoyed or irritable	◯	◯	◯	◯
Feeling afraid as if something awful might happen	◯	◯	◯	◯

Survey Progress

Over the past TWO WEEKS, how often you have been bothered by the following? (Patient Health Questionnaire - PHQ-9)

	Not at all	Several days	More than half the days	Nearly every day
Little interest or pleasure in doing things?	○	○	○	○
Feeling down, depressed, or hopeless?	○	○	○	○
Trouble falling or staying asleep, or sleeping too much?	○	○	○	○
Feeling tired or having little energy?	○	○	○	○
Poor appetite or overeating?	○	○	○	○
Feeling bad about yourself - or that you are a failure or have let yourself or your family down?	○	○	○	○
Trouble concentrating on things, such as reading the newspaper or watching television?	○	○	○	○
Moving or speaking so slowly that other people could have noticed? Or been so fidgety or restless that you have been moving a lot more than usual?	○	○	○	○
Thoughts that you would be better off dead, or thoughts of hurting yourself in some way?	○	○	○	○

Survey Progress

Over the last TWO weeks, how has the COVID-19 pandemic impacted your everyday thinking? (Impact of Event Scale - IES-6)

	Not at all	A little bit	Moderately	Quite a bit	Extremely
I thought about it when I didn't mean to	○	○	○	○	○
Other things kept making me think about it	○	○	○	○	○
I was aware that I still had a lot of feelings about it, but I didn't deal with them	○	○	○	○	○
I tried not to think about it	○	○	○	○	○
I felt watchful or on guard	○	○	○	○	○
I had trouble concentrating	○	○	○	○	○

Survey Progress

For each statement, mark the box that most accurately reflects your response: (Abbreviated Maslach Burnout Inventory)

	Every day	A few times a week	Once a week	A few times a month	Once a month or less	A few times a year	Never
I deal very effectively with the problems of my patients	◯	◯	◯	◯	◯	◯	◯
I feel I treat some patients as if they were impersonal objects	◯	◯	◯	◯	◯	◯	◯
I feel emotionally drained from my work	◯	◯	◯	◯	◯	◯	◯
I feel fatigued when I get up in the morning and have to face another day on the job	◯	◯	◯	◯	◯	◯	◯
I've become more callous towards people since I took this job	◯	◯	◯	◯	◯	◯	◯
I feel I'm positively influencing other people's lives through my work	◯	◯	◯	◯	◯	◯	◯
Working with people all day is really a strain for me	◯	◯	◯	◯	◯	◯	◯
I don't really care what happens to some patients	◯	◯	◯	◯	◯	◯	◯
I feel exhilarated after working closely with my patients	◯	◯	◯	◯	◯	◯	◯

Survey Progress

Is there is anything else that you would like to tell us about the impact of the COVID-19 pandemic or regarding supports that you feel are useful for well-being?

____
